# CINGLE-trial: cochlear implantation for siNGLE-sided deafness, a randomised controlled trial and economic evaluation

**DOI:** 10.1186/s12901-015-0016-y

**Published:** 2015-05-23

**Authors:** Jeroen PM Peters, Alice van Zon, Adriana L Smit, Gijsbert A van Zanten, G Ardine de Wit, Inge Stegeman, Wilko Grolman

**Affiliations:** 1 Department of Otorhinolaryngology and Head & Neck Surgery, University Medical Center Utrecht, PO BOX 85500, 3508 GA Utrecht, The Netherlands; 2 Brain Center Rudolf Magnus, University Medical Center Utrecht, Utrecht, The Netherlands; 3 Julius Center for Health Sciences and Primary Care, University Medical Center Utrecht, Utrecht, The Netherlands

**Keywords:** Single-sided deafness, Unilateral hearing loss, Contralateral routing of sound, Bone conduction device, Cochlear implantation, Speech perception in noise, Sound localisation, Tinnitus, Quality of life, Economic evaluation

## Abstract

**Background:**

Individuals with single-sided deafness (SSD) have problems with speech perception in noise, localisation of sounds and with communication and social interaction in their daily life. Current treatment modalities (Contralateral Routing of Sound systems [CROS] and Bone Conduction Devices [BCD]) do not restore binaural hearing. Based on low level of evidence studies, CROS and BCD do not improve speech perception in noise or sound localisation. In contrast, cochlear implantation (CI) may overcome the limitations of CROS and BCD, as binaural input can be restored. Promising results have previously been achieved on speech perception in noise, sound localisation, tinnitus and quality of life.

**Methods and design:**

A single-center Randomised Controlled Trial (RCT) was designed to compare all treatment strategies for SSD. One hundred and twenty adult single-sided deaf patients (duration of deafness >3 months and maximum 10 years; pure tone average at 0.5, 1, 2, 4 kHz, deaf ear: threshold equal to or more than 70 dB, better ear: threshold of maximum 30 dB) will be included in this trial and randomised to CI, ‘first BCD, then CROS’ or ‘first CROS, then BCD’-groups. After the trial period, patients in the two latter groups may choose with which treatment option they continue. Outcomes of interest are speech perception in noise, sound localization, tinnitus and quality of life. These outcomes will be measured during a baseline visit and at follow up visits, which will take place at 6, 12, 18, 24, 36, 48 and 60 months after onset of treatment. Furthermore, an economic evaluation will be performed and adverse events will be monitored.

**Discussion:**

This RCT allows for a comparison between the two current treatment modalities for single-sided deafness and a new promising treatment strategy, CI, on a range of health outcomes: speech perception in noise, sound localization, tinnitus and quality of life. Additionally, we will be able to answer the question if the additional costs of CI are justified by increased benefits, when compared to current treatment strategies. This study will inform health policy makers with regard to reimbursement of CI.

**Trial registration:**

Netherlands Trial Register (www.trialregister.nl): NTR4580.

## Background

Individuals with single-sided deafness (SSD) have problems with speech perception in noise and localisation of sound [1, 2]. With only one functional ear, they cannot benefit from binaural summation (redundancy of auditory input) [3] and squelch effects (ability of the brain to separate sound and noise signals from spatially separated sources) [1, 2]. Moreover, the head acts as an acoustic barrier and thus attenuates signals from the deaf side going to the better ear, known as the geometric head shadow effect [4]. Patients suffering SSD experience problems in their daily life in social interaction and communication [5].

Current treatment modalities for patients with SSD are Contralateral Routing of Sound (CROS) systems and Bone Conduction Devices (BCD). A CROS conducts signals from the hearing field of the poor ear via a wire (or FM/Bluetooth) to an output transducer in the ear canal of the better ear such that sound awareness is restored. A BCD transfers signals from the hearing field of the poor side to the better hearing ear by vibration of the skull bone via a titanium implant. A trial with a BCD can be performed by attaching the BCD to a tight headband. Theoretically, CROS and BCD can alleviate the head shadow effect. However, neither modality can restore binaural hearing. A recently published review found that there are no high level of evidence studies comparing CROS and BCD for single-sided deafness [6]. The authors of the review could only include studies with low to moderate levels of evidence, and they found that CROS and BCD did not improve speech perception in noise or sound localisation, although patients did benefit in speech communication subjectively [6].

A new treatment option for single-sided deafness, cochlear implantation, may overcome the limitations of CROS and BCD. Since cochlear implantation restores auditory input on the impaired side, binaural input can be restored. Speech perception in noise and sound localisation improved in patients with single-sided deafness treated with a cochlear implant (CI) [7–9]. Furthermore, cochlear implantation may reduce tinnitus and improve quality of life [10]. However, the quality of the studies included in these reviews was suboptimal: sample sizes were small, study designs were case series and they were prone to selection bias [11].

The reviews on both current and new treatment options for single-sided deafness make clear that high level of evidence studies are warranted. Therefore, we initiated a Randomised Controlled Trial (RCT) comparing CROS, BCD and CI for SSD. Outcomes of interest are speech perception in noise, sound localization, quality of life and tinnitus. Finally, an economic evaluation will be performed. Since cochlear implantation is more expensive than current treatment options, it is important to know whether the promising results of cochlear implantation outweigh the additional costs.

## Methods

### Study objectives

The main objective of our study is to compare CROS, BCD and CI in patients with SSD evaluating speech perception in noise, sound localization, quality of life and tinnitus. The second objective is to perform an economic evaluation and to evaluate adverse events in all groups.

### Study design

**Fig. 1:**
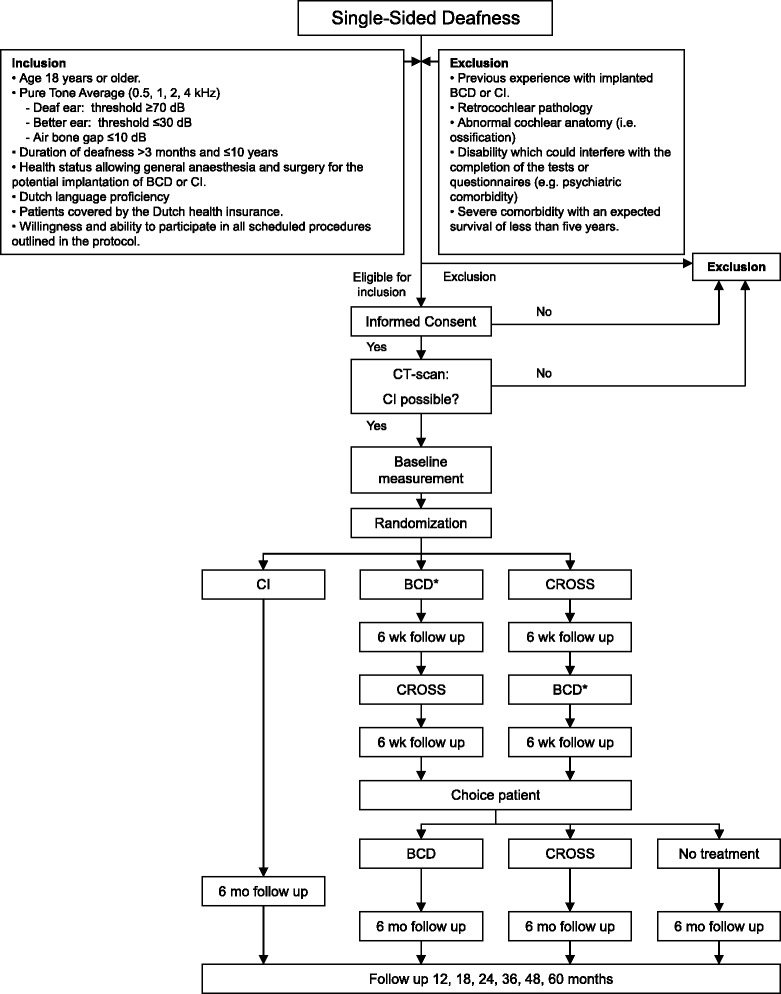
Flow diagram of CINGLE-trial. Abbreviations: BCD = Bone Conduction Device (* indicates trial period on headband), CI = Cochlear Implant, CROS = Contralateral Routing of Sound system, mo = months, wk = weeks

In this RCT patients are randomised in three groups. For a schematic overview of the study, see Fig. 1.

We will now discuss all consecutive steps in the study design as included in this study protocol. This protocol is reported according to the SPIRIT Statement, an international guideline on the reporting of study protocols [12].

### Study population

The study population consists of patients who present with SSD at the outpatient department of our tertiary referral center, the University Medical Center Utrecht, Utrecht, The Netherlands. They must meet the following criteria to be eligible for the study.

#### Inclusion criteria

- Age 18 years or older
- Audiometry (Pure Tone Average [PTA] at 0.5, 1, 2, 4 kHz)
  - ○ Deaf ear: threshold of ≥70 dB
  - ○ Better ear: threshold of ≤30 dB
  - ○ Air bone gap ≤10 dB (to ensure normal middle ear function)
- Duration of deafness >3 months and ≤10 years
- Health status allows general anaesthesia and surgery for the potential implantation of BCD or CI
- Dutch language proficiency
- Coverage of Dutch health insurance
- Willingness and ability to participate in all scheduled procedures outlined in the protocol

The minimum duration of deafness is 3 months, since that is the time in clinical practice to await the natural course of sudden deafness. The maximum duration of deafness is up to 10 years, since degeneration of the auditory nerve may occur.

#### Exclusion criteria

- Previous experience with implanted BCD or CI
- Retrocochlear pathology
- Abnormal cochlear anatomy (i.e. ossification)
- Comorbidity
  - ○ which could interfere with the completion of the tests or questionnaires (e.g. psychiatric)
  - ○ with an expected survival of less than five years

If eligible for inclusion, an Informed Consent (IC) form will be signed by patient and researcher. Only after IC, a CT-scan of the mastoid will be made, if none is available yet, to assess cochlear anatomy and check if no contraindications to cochlear implantation (e.g. ossification) exist. Since the anatomical situation must allow cochlear implantation (and thus randomisation), CT-scans will be performed prior to randomisation.

### Randomisation and interventions

A web-based randomisation tool (Julius Center, University Medical Center Utrecht, Utrecht, The Netherlands) will be accessed via a computer by one of the members of the research team. Patients will be randomised into groups A, B and C (see Fig. 1), using a block size of 8 and a ratio of 2:3:3 for groups A:B:C and stratified for age (<45 years, ≥45 years). Blinding and concealed treatment allocation is not possible in our study design.

In group A, patients will be implanted with a CI from Cochlear Ltd., (type CI422). A retro-auricular incision is made to expose the mastoid. The electrode is inserted via a posterior tympanotomy and round window implantation. Intraoperatively, normal functioning of the device is checked by measurement of impedance and neural response telemetry. Four weeks after implantation, the CI will be activated by an experienced audiologist. In the rehabilitation phase, patients will be encouraged to use the CI each day and will be trained by experienced speech and language therapists. Patients are instructed to train the cochlear implant ear using an International Speech Test Signal (ISTS) noise [13] via an insert earphone to mask the better ear.

In the Netherlands, a trial period with both CROS and BCD is standard clinical care. Patients in groups B and C try both devices for 6 weeks: group B starts with the BCD (type: BP110, Cochlear Ltd.), then CROS (Phonak Audeo Q50-312T and CROS H_2_O), whereas patients in group C start with the CROS and then try the BCD. The reversed order is implemented to correct for the order effect: patients judge their second hearing aid based on experiences with the first hearing aid. After these two trial periods, patients may, according to clinical practice, choose which of both treatments they like best. When they choose a BCD, the implant and abutment will be surgically implanted, and after six weeks mounted with a BCD (type: BAHA 4 system, Cochlear Ltd.). When they prefer CROS, the patient is referred to standard clinical care where the CROS will be adjusted. Patients can also opt for no treatment if none is preferred, according to standard clinical health care.

### Sample size

To detect a clinically relevant difference of 5 dB signal-to-noise ratio (SNR) (standard deviation 5 dB) between the groups on the primary outcome (see *Outcomes*), with an alpha of 0.05 and a power of 95%, 27 subjects per group are needed. To compensate for potential dropouts, a 10% margin is implemented, resulting in 30 patients in group A. Initial group distribution will be n = 45 for groups B and C. These groups are bigger, since previous studies describe that only 45% of BCD-on-headband users are satisfied after their trial period and opt for a BCD implantation (n ~ 45) [14]. Therefore, we will include more patients in groups B and C than in group A. Patients not choosing a BCD can opt for a CROS (approximately 60% of remaining patients, n ~ 30). The rest of the patients (n ~ 20) will probably prefer no treatment; they will be followed up to assess the natural course of single-sided deafness.

Approximately 25–30 patients per year present with SSD at the otorhinolaryngological outpatient department of our tertiary referral center. We will actively invite audiologic centers in the neighborhood to refer patients to our clinic. Therefore, we expect the inclusion period to last for ~3 years.

### Outcomes

The following outcome measurements will be recorded during baseline visit (one condition, i.e. ‘no device’) and follow up visits at 6, 12, 18, 24, 36, 48 and 60 months ‘device on’. During all audiometric tests, patients will be instructed not to move their heads to improve speech perception or sound localisation. Head movements will be checked by the researcher conducting the experiments. Patients will not receive feedback on their performance on audiometric tests. All experiments will be performed by researchers following the same protocol procedures.

#### Primary outcome measure

**Fig. 2:**
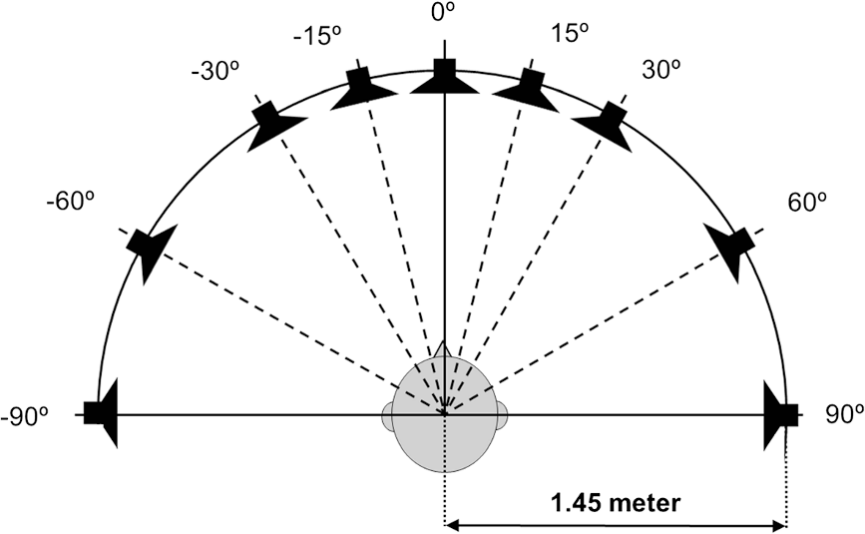
Set-up York Crescent of Sound. The patient is positioned in the center of an arch of loud speakers (-90 to 90°) at head level with a radius of 1.45 m [16]

Our primary outcome is the performance on speech perception in noise, measured with the *Utrecht Sentence Test with Adaptive Randomized Roving Levels* (U-STARR) [15]. In short, the U-STARR is designed to determine a patient’s ability to understand speech in a noisy environment (signal from front, noise from front: S_0_N_0_. See Fig. 2, test set-up York Crescent of Sound [16]). A sentence is considered to be understood correctly when ≤2 words are repeated incorrectly. The noise level starts at +20 dB (roving 65–75 dB SPL). When the sentence is repeated correctly, the level of the noise increases for the next sentence. Noise is presented 500 ms before the start of the sentence and ends 500 ms after the sentence. Sentences used are traditional Dutch sentences from everyday life [17]. The test provides a critical SNR at which 50% of sentences is understood correctly (in dB).

#### Secondary outcome measures

##### Speech perception in noise

Speech perception in noise is measured in two more configurations: S_−60_N_+60_ and S_+60_N-_60_ (Fig. 2). Again, Dutch sentences are used in the same audiometric set-up as the U-STARR [15, 17] leading to a SNR (in dB).

##### Sound localisation

The ability to localise sounds is also measured with the York Crescent of Sound set-up [16]. The stimulus (sentence by female speaker: ‘Hello, what’s this?’) is presented in quiet at a roving level of 55–65 dB SPL in three configurations: 5 boxes separated by an angle of 15° (box -30°, box -15°, box 0°, box +15° and box +30°), 5 boxes separated by an angle of 30° (box -60°, box -30°, box 0°, box +30° and box +60°) and 3 boxes separated by an angle of 60° (box -60°, box 0° and box +60°) (Fig. 2). The patient must indicate from which box the stimulus came. The test outcome is a percent correct score.

##### Tinnitus

Tinnitus burden will be assessed using three questionnaires:

- Tinnitus Handicap Inventory (THI): a 25-item questionnaire with statements/questions about tinnitus burden [18]. Possible answers are ‘Yes’ (4 points), ‘Sometimes’ (2 points) and ‘No’ (0 points), resulting in a maximum score of 100, representing a maximum burden of tinnitus. The inventory is divided in a functional, emotional and catastrophic subscale.
- Tinnitus Questionnaire (TQ): a 52-item questionnaire consisting of 5 subscales: emotional and cognitive distress, intrusiveness, auditory perceptual difficulties, sleep disturbances and somatic complaints [19, 20]. Possible answers are ‘Yes’ (2 points), ‘Sometimes’ (1 point) and ‘No’ (0 points). Of the 52 questions, 38 constitute a final TQ-score on the validated Dutch version of the TQ [21]. Tinnitus can be graded mild (TQ 0–16), moderate (TQ 18–34), severe (TQ 34–56) and catastrophic (TQ >58).
- Tinnitus Burden Questionnaire (TBQ): this is a self-developed questionnaire assessing various aspects of tinnitus burden. It consists of 12 visual analogue scales (VAS), ranging from ‘0’ (no tinnitus burden) to ‘10’ (maximum tinnitus burden).

##### Quality of life

Participants will be asked to fill in several questionnaires, each assessing different parts of QoL.

- Speech, Spatial and Qualities of hearing scale (SSQ): this questionnaire assesses three domains of hearing: 1) Speech: consists of 15 questions about the ability to separate speech from competing noise in a wide range of listening contexts; 2) Spatial: consists of 17 questions to assess the ability to locate sound sources and their direction of movement; 3) Quality: consists of 19 questions that assess naturalness and clarity of sounds. The responses are given on a VAS ranging from 0 (not able to) to 100 (perfectly able to) [22].
- Abbreviated Profile for Hearing Aid Benefit (APHAB): this 24-item questionnaire documents the outcome of a hearing aid [23]. The questionnaire yields scores on subscales for ease of communication, listening under reverberant conditions, listening in background noise and aversiveness of sound.
- Glasgow Benefit Inventory (GBI): a measure of patient benefit developed especially for otorhinolaryngological interventions [24]. The inventory is validated to measure outcomes on health status after otorhinolaryngological procedures. It measures QoL in three domains: social, general and physical. The domains score on a scale of -100 to 100 (minimum versus maximum benefit, respectively).
- Hospital Anxiety Depression Scale (HADS): a screening tool for anxiety and depression in non-psychiatric clinical populations [25]. We use the HADS to measure baseline depression symptoms, which may confound/bias the results of, for instance, tinnitus burden.
- A VAS consisting of two questions (quality of life, quality of hearing).
- Time Trade Off (TTO): comprises one question about how many years of their lives patients would sacrifice for living with perfect hearing for the rest of their lives. TTO (%) = ((life expectancy – number of years to give up for perfect hearing) / life expectancy) * 100. This question is generally considered a difficult question, so it will not be presented on paper, but asked during the baseline and follow up visits.
- EuroQoL5D (EQ5D): is a measure of general health status [26]. It contains 5 questions on mobility, self-care, daily activities, pain/complaints, anxiety/depression and a scale to denote general quality of life (VAS 1–10). We will use the Dutch EQ5D tariff [27].
- Health Utilities Index 3 (HUI3): this is a generic quality of life questionnaire consisting of 8 domains: vision, hearing, speech, ambulation, dexterity, cognition, emotion and pain [28].

##### Economic evaluation

The latter three questionnaires can be used to calculate utility. Utility reflects the value that is attached to health status. Utility values are important for the calculation of Quality Adjusted Life Years (QALYs), which serve as the denominator for the Incremental Cost Utility Ratio (ICUR). The ICUR is calculated as the incremental costs of cochlear implantation as compared to current treatments (numerator) divided by the incremental effects in terms of QALYs. Costs will be measured from a societal and health care perspective. Both direct health care costs and indirect non-health care costs will be incorporated in the analyses. Both categories of costs will be quantified using a cost diary. This diary is completed on a monthly basis the first 2 years and on a quarterly basis the last three years. This diary assesses costs related to hospitalisation, surgery, blood tests, complications (direct health care costs) and sick leave, time and travel costs (indirect health care costs). Unit prices for volumes of resources use will be taken from the Dutch guidelines for costing research in health economic evaluations, as issued by the National Healthcare Institute [29].

Incremental Cost Utility Ratios, comparing CI with CROS and BCD, will be estimated using bootstrapping. Cost-effectiveness planes and a cost-effectiveness acceptability curve will be plotted to visually represent the results of the economic evaluation.

In addition to these outcome measurements at the previously specified baseline and follow up visits, we will also objectify the experiences of the patients in Groups B and C in the trial periods with CROS and BCD (see Fig. 1). To minimize patient burden, only the APHAB, GBI and SSQ questionnaires will be administered to evaluate these trial periods.

### Statistical analysis

Baseline characteristics per group will be described as means and standard deviations. Differences between the three groups will be analysed using the Kruskal Wallis test.

The data of our primary outcome are quantitative and will be presented as continuous variables. Between-group mean differences, rate differences and rate ratios with 95% confidence intervals will be calculated. Again, the Kruskal Wallis test will be used to analyse differences between the groups.

The secondary outcomes contain both categorical and continuous outcomes. Analyses of between-group differences will be performed with Chi-square-tests for categorical outcomes and Kruskal Wallis tests for continuous outcomes. Within-subject comparisons will entail differences of mean values. These will be analysed using paired t-tests for continuous measures.

Major test intervals are the same in all study groups (baseline and 6, 12, 18, 24, 36, 48, 60 months follow up; 6 and 12 weeks for group B and C). Missing values will be imputed using multiple imputation. All analyses will be performed on an intention-to-treat basis. A significant result is defined as a *p*-value < 0.05. Statistical package SPSS will be used for statistical analyses of the data.

Data will be presented according to the Consolidated Standards of Reporting Trials (CONSORT) Statement, an international guideline on adequate reporting RCTs [30, 31].

### Safety

This study will be conducted in accordance with the most recent version of the Declaration of Helsinki (Fortaleza, 2013), good clinical practice guidelines and the Medical Research Involving Human Subjects Act of the Dutch government. The research protocol was approved by the Institutional Review Board (IRB) of the University Medical Center Utrecht (NL45288.041.13; version 3, April 2^nd^, 2014).

All cases of serious adverse events will be reported to the local IRB and adequately followed up. An independent monitor (Trial Form Support BV, Zaltbommel, The Netherlands) is appointed to check trial quality (completeness of IC, validity of data etc.) twice a year. All patient data will be stored on a password protected computer in a lockable room. In the same room the signed Informed Consent forms will be kept in a locked cabin.

### Trial status

The trial is currently in recruitment phase.

## Discussion

Patients suffering SSD experience problems with speech perception in noise and localisation of sound and in social interaction and communication [5].

Current treatment modalities do not restore binaural hearing for patients with SSD. A recently published review concluded that there are no high quality studies comparing CROS and BCD for single-sided deafness [6]. With this limited level of evidence, CROS and BCD did not improve speech perception in noise or sound localisation. Patients did benefit in speech communication subjectively. Cochlear implantation may overcome the limitations of CROS and BCD, as binaural hearing can be restored. Promising results have previously been achieved on speech perception in noise, sound localisation, tinnitus and quality of life [7–11].

The current study is the first high level of evidence trial to be conducted to effectively compare all treatment strategies for single-sided deafness. One hundred and 20 adult single-sided deaf patients will be included in this trial and randomised to CI, BCD-CROS or CROS-BCD groups (Fig. 1). Outcomes of interest are speech perception in noise, sound localization, quality of life and tinnitus. Finally, an economic evaluation will be performed to answer the question if the additional costs of cochlear implantation are justified by increased benefits compared to current treatment strategies.

## Abbreviations

APHAB: Abbreviated profile of hearing aid benefit questionnaire
BCD: Bone conduction device
CI: Cochlear implant
CINGLE: Cochlear Implantation for siNGLE-sided deafness
CONSORT: Consolidated standards of reporting trials
CROS: Contralateral routing of sound
CVC: Consonant-vowel-consonant
dB SPL: decibel sound pressure level
EQ5D: Euro-QoL 5D questionnaire
GBI: Glasgow benefit inventory
HADS: Hospital anxiety distress scale
HUI3: Health utilities index (version 3) questionnaire
IC: Informed consent
ICUR: Incremental cost utility ratio
IRB: Institutional Review Board
ISTS: International speech test signal
PTA: Pure tone average
QALY: Quality adjusted life year
QoL: Quality of life
RCT: Randomised controlled trial
S_−60_N_+60_: Speech coming from -60°, noise coming from +60°
S_0_N_0_: Speech and noise coming from front (0° azimuth)
S_+60_N_−60_: Speech coming from +60°, noise coming from -60°
SNR: Signal-to-noise ratio
SPIRIT: Standard protocol items: recommendations for interventional trials
SSD: Single-sided deafness
SSQ: Speech, spatial and quality of hearing scale
TBQ: Tinnitus burden questionnaire
THI: Tinnitus handicap inventory
TQ: Tinnitus questionnaire
TTO: Time trade off questionnaire
U-STARR: Utrecht-sentence test with adaptive randomized roving levels
VAS: Visual analogue scale
